# Supplementary material for: Molecular identification and expression patterns of odorant binding protein and chemosensory protein genes in Athetis lepigone (Lepidoptera: Noctuidae)
Source: PeerJ. 2017 Mar 30;5:e3157. doi: 10.7717/peerj.3157 (PMC5376112; doi:10.7717/peerj.3157)
Supplement: Table S1 [file peerj-05-3157-s002.pdf]

Table S1. Amino acid sequences of AlOBPs and AlCSPs obtained in the study.

OBP

>AlPBP1
MADPRWQFTRFVCVMFMASSVMASKELLTKMSTGFTKVLDACKTELNAGDHIMQDMY
NFWKEEYELVNRDLGCMVLCMANKLELIGDNQKLHHGKAEEFAKSHGADDGQAKQLV
ALVHDCENQHQGVEDACSKMLEVSKCFRTKIHELKWAPSMEVVMEEIMAAANA
>AlPBP2
MALHPPVTMTVRLALVVIASLVIA
VESSQEIMKTLSLNFAKPLQDCKKEMDLPDTVTTDFYNFWKEGYEFTNRHTGCAIMCLSS
KLELLDQELKLHHGKAQEFAMKHGADENMAKQLVEMIHSCAESTPDAADDPRMKALHV
AQCFKNKIHDLKWAPSIELIMGEVLAEL
>AlPBP3
MGRCSMFLVMLIMAVAVWKVEP
SKDAMKYITSGFVKVLEECKKELNMDEHILADLFHFWKLEYSLLDRDTGCVIICMSKKLD
LLDENGRMHHGNAQEFALKHGAGEEVAAKIVTIIHECEKKFERDDDECLRVLEVAKCFRS
GIHELDWQPKVQTIVSEVLTEI
>AlGOBP1
MQAVRALVLLAAAGVLRA
DVLVMKDVTLGFGQALDKCRKESDLTEEKMEEFFHFWREDFKFEHRELGCAIQCMSNYF
NLLTDSSRMHHGNTEKFILSFPNGEVLARQMVELIHSCEKQFDHEADHCWRILHVAECFK
SACVAHGIAPSMEMMMTEFIMESEAR
>AlGOBP2
MTPKYCMLVVVVAAVASSVMA
TQEVMSHVTAHFGKALEECREESGLSAEILEEFQHFWREDFEVVHRELGCAIICMSNKFSL
LKDDSRMHHVNMHDYVKGFPNGEVLSARLVELIHNCEKQYDSLPDDCDRVVKVAACFK
VDSKAAGIAPEVAMIEAVMEQY
>AlOBP1
MECNNEHPITPKEMLLLKANKIPESESAKCFVACIFKRTGMLNSKGMYDAAASIALTEKDF
ADDPKKLENSKKLLESCKNVNDEPVKDGEKGCERSVHLHKCIVDTAAQLGIKLPN
>AlOBP2
MFKYFIYCVFIVSASHA
DLLSQKENKGATLKPLSVCCDIPELGDPKFLAKCSNPKLPGPCNDVQCVFEESGFLTDRNT
LNKEAYKAHLRKWEENNKGWTVAVDKAIADCVDNDPRQHLDVPCKAYDVFTCTGIAML
KKCPEAAWKC
>AlOBP3
MTMKQIRNTGKMMRKSCQPKNNVEDEKIDPIADGVFIDEKEVKCYMACIMKMANTIKN
GKINFDAAIKQADLLLPDEVKEPAKEAILACKKAADHKDICDASFHLTKCIYNQNPGIFYF
P
>AlOBP4
MKSFVVFCVVVAGIYA
ADVPLPAAQQEKAKAISAECVKESGVSKAVLADALKGTLADDEGLKKFTLCFFHKAGIV
DGQGALNIDVALAKLPPGVDKDNAKSVLEGCKSKTGKDTAEKVFEILKCYHMGVKNHV

LFAGI
>AlOBP5
MSKFTCLVLCLVAASISRVYA
GEEEKAAFREAIKPIIEECSNDHGVSVEDIESAKAAGSADGIKPCFLGCVYKKAEVINSKGE
YDVDTALSKLKTFVSDDAKYAKLAEVGKKCASVNEKAVSDGDAGCERGALLTACFLENK
GEALI
>AlOBP6
MWFRTIVLVAALAAARA
VEMDEDMAELARMVRENCIAETGADVALVEAVNGGADLMPDDKLKCYIKCTMETAGM
MGDGEVDVEAVLALLPPELAARNGPALNACGTQRGADDCDTAWKTQVCWQSANKADY
FLI
>AlOBP7
MIRSCSLVLAVLVQVLLGQA
QEPAPFPQFQDRIPRHCLAPPPGINLHTCCPIPNLYPDEVMESCGIEKVRQDNPNAPPKPRG
PPKAPCKEGICLMQHADLLLANQSVDYEKLRSFIDHWAESNPDFSEAILAAKEICAKDGGP
SGPPVCEQDKIFFCLTSNILWNCKLRDLDGNSGCSILKAHMDECRPHFLKRKELEEQNGQ
>AlOBP8
MYHLYISVFVCCVFSISVKA
SSLDELKMKYVELILECSNSYPITRDDMSLLRRKIMPDSESAKCLFACVYKKAGMMNEHG
QLSVQGVNEMTRKYLADDPEKIKKSEEFTQACESVNDVEVSDGERGCDRAALIFKCTVE
KSPDFDLF
>AlOBP9
MKTLMVFAACILLAQA
LTDEQKEKLKKHRTECLTETKVDEQLVNKLKGGDYKTESEPLKKYALCMLMKSELMTKD
GKFKKDVALAKVPNAADKPMVEKLIDACLANKGNTPQQTAWNYVKCYHEKDPKHAIFL
>AlOBP10
MKWLDGSSVDKAKLTTFFEQFEKDHAEWASAVQQVKTVCLGSELKAQGVFLNCPVYDV
MHCILGAFVKHATPSQWSTEASCSYARAYAADCPVCPN
>AlOBP11
MTDFACLILCAVVVNISIAYA
DESTRARQDEVSAFLRECAKEYGVEEQSIDQAAMSQDVTLVNSCFWACVLKKTGFLNDK
GEYDMKTGMMYVKEVVPQESTYKNLEDVAKLCEAVKDKPVNDGEAGCERGAQVVDCF
LKQMETQMKMAAHQK
>AlOBP12
MFRWELPVVLCFIAAALGGKEKPVFS
DEIKEIIQTVHDECVAKTGVAEEDITNCEKGIFKEDPKLKCYMFCLMEEASLVDDDDVVDY
DMLVSLIPDEYYERTTKMIFSCKHLDTPDKEKCQRAFEVHKCSYEKDPDLYFLF
>AlOBP13
MVRQIGLLLCSLCVFGISLS
DSAISADSESRCRNPPTAPQKIERVITLCQDEIKLSILREALDVIKEEHTMPAQRRRDKREVP
FTHDEKRIAGCLLQCVYRKVKAVDGYGFPTLEGLVGLYSDGVNERGYFMAVLEASRECL
MKNHDKFSRTVPMDNGRNCDISFDIFECISDRIGEYCGTAGL
>AlOBP14
MFKFCAFLLFWVASCYA

APGAGTYCGETPDVIYNCLSAPKLVSSEISNKCTGAKYSNECDKLTCVFREAKWLNDATV
DKAKLTTYFEQFERDHAAWSPAIQHVKTACLGSELKPQGVNLNCPAYDIMHCALSSFIKH
ATPSQWSTAASCTYPRAYATDCPVCPNDCFSPQVPIGSCNACYLQPPAA
>AlOBP15
MFKSSAIFLCCLFFCALTPYLTYA
MTAEQKAQIHAHFEAIGMSCNKDSTMITSEDIADLRAKKIPSGPNAPCFLACMMKQIGVM
DESGMLQKETLLEMAKKIFDDAEELKIIEDYLHSCAHINGESVSDGAAGCDRAMLAYKC
MTENASQFGIEV
>AlOBP16
MFVYGRLSFAAVLLCLGCTYA
ITKEDEASLKQALHPHVMECAEEFGITPEQFEEAKKKENAKHMDPCFLSCMMKKAEFLD
SDGKIDFEKTVSFAKDHELSEKAVKFFETVGEECAKVNDDEVSDGEKGCERAKLLFHCVH
EIKKKMAE
>AlOBP17
MVDAVVLLMLLPAWAACSG
EGNIKLLEDEVAVALKSCTYPDDTVVSKEPVSKERQRRGSDDTYDGSPRIDNNMKEGNRY
SHERRNTNNSGDQIQVFNATDYDYEGYGTGSNGEKLLTSVPRPASPSNNVNNNNTSRTRR
SEPLLNKQDLDQCLSQCVFANLQVVDTRGIPREAELWNKVQSSVTSQQSRSALHYQIRAC
FQELQSEAEDNGCSYSNKLEKCLMLRFSDRKVDGKASTQKPASTEQS
>AlOBP18
MSKFTCIVLCVVAASLVKVSHA
VTEEEKAAFREAMAPVIAECSEEHGVSESDIQAAKEAMSADAIKSCFLGCVMKKTEAVDA
KGMFDADAGLSKIRKYVNSDDDFAKFEKIGKLCMSVNDKEVSDGEAGCERAKLVLACFL
EHKADIPF
>AlOBP19
MDDMGMISPEKARENAKKVFKGSDEHLKNVDKIMDTCSKVNQQTTSDGNKGCDRAKL
AFGCFTENAPKVSC
>AlOBP20
MVKMARLLLSMVLTITTVALTQS
ASTSMKDAATKEAMSTTMADMGSGIDTLDVNVLDVMSACNESFRIEQAYIQSMNETGSFI
DETDKTPKCFIRCVFENVGIVSEDGKQFNPARAAVIFAGERNGKPMDDIGDMTAMCAADR
QESCLCDRSYQFLRCLMSMEIERYEKA
>AlOBP21
MFKSSVVYFAILAVFFKNALA
ITDEQKQQIQTKVVAVGAECLKEYPLSIDDLASFKSRVFPEGENAGCFSACIFNKLGLFDDK
GTWSHVTALEHAKKVFDDEEVLKNIEAFLTTCAKVNEEEVKDGEKGCDRAKLAFDCFVK
NYEQLGFNFDF
>AlOBP22
MVYSSNIFLFTVSLIFMLNSSYVSS
MTREQIKNSGKLIKKTCSAKNGLTEDQVKDVDKGKFIEEKNFMCYIACVYKMGQSIKGN
TINHDMMVRQVEMMFPSEMKTPVKAAIEHCRGVAKKYKDVCEASYWTAKCIYEFDPAN
FMFP
>AlOBP23
MWNFIILFVAICSCVYG

LTEQELKVEFTKLIMKCNKDSEVDMMELVQLQSYVVPTKTATKCVLACAYKAANVMNA
QGLYDIDHAYKVAEMMKNGDEKRVINGKKMADVCVKVNDVKVSDGEKGCDRAALIFK
CTVDNAPKFGFKL

CSP

>AlCSP1
MKAVFLLCALVVVVSA
RPEAQYTNKYDNVDLDEIIQNRRLLVPYIKCGLDQGKCTPDGKELKSHIKEALETYCAKC
TDVQRDGTRRVIAHLINHEPEYWRELSAKYDRDGKFARKYEDELRTVA
>AlCSP2
MKLLIILALVAAAFA
RPDDAHYDSKYDNFDVDELIQNERLLKAYAHCIIGDGKCTPEGNEIKGWTPEAVQTSCGK
CTEKQKVLVAKCIKAIREKLPEEFELLVKKNDPEGKHKEGLKSFLEKYA
>AlCSP3
MNALLVAVFALVASSALA
YDEIYDKIDVDKILGDDALFSAYINCMLDKGDCSVEHSADFRKLLPEVIATSCAKCSPIQR
QNVRKTVKALSEKRPDEFAEFRTKYDPKGEYEKSFTEFVMGTD
>AlCSP4
MRAVLLMCVFVYAVVA
QDVNDMLNLPKYDSRYDYLDVDAIFTNKRLVRNYVDCLINAVRCTPEGKALKRILPEALR
TKCVRCTERQQRTAVKVIKRLKNEYPDEWAKLSSRWDPTGDFTRYFEEFLAKENFNTIPGS
GSAIPTSSPLVPPRVTTMPATVATPAPGPTEPAPAQPAIFNRFGDDDEVMMGSPSSAGMTPRP
MTQATTRPTTTMRPTTTMRPITSKPLSPRPTMMTWSGAATNTQPTRFPLRPVAELPIPYSTA
ITLIDQIGYKIIKTTELVTDLLKHTVRAVVGR
>AlCSP5
LKTGCSKCTARQRGGIKKVMGHLIKHEPEYWARAVDKYDPDRVYTKKYEQEVYSWQ
>AlCSP6
MHSTIAMLLLVYLTIQSNA
TETSTYTTKYDGIDLDEILNNDRLLTGYVNCLLDIGPCTADGKELKQNLPDAIENDCKKCT
EKQREGSERVMHYIIDNRPDDWVKLEDKYNTDGSYKLKYLASKLTEADKETNVTTSEEN
TKNVSKESSKE
>AlCSP7
MKVLVVLPVLVAFAAAAAA
ELSPAELSMLEAFDYETLLANKELSQKLFDCMLEKGDCGEYKQVADLSMKTLQSKCAEC
TPAQKAKYENVLKQLKEKYEPVYNELLKKAGATQKT
>AlCSP8
MKVIVAIALLCIVAVAWG
KPAGTYTDKWDHINVDEVLESQRLLKAYVDCLMDRGRCTPDGKALKETLPDALEHECSK
CTEKQKTGSDKVIRHLVNKRPDLWKELSTKYDPDNIYQERYKNKIEAVKQ
>AlCSP9
MKFVLLLCVMVAAVLA

EDKYTDKYDNLNVDEILTNKRLLEAYVNCVLEKGKCTAEGKELKEHLQDAIETGCKKCT
EAQEKGAQKVIDYLIKNELGYWRELTDKYDPTGTWRKTYEDRAKAAGIKIPE
>AlCSP10
MNYLVLSVVVTLAAFVAA
ETYTDRYDHINIDEIIDNRKLLVPYIKCTLDQGRCTPEGRELKAHIKDAMQTSCSKCTPKQ
RKAARKVVKHIRAKEQDYWKQIIAKYDPNDEYKENYETFLETTD
>AlCSP11
MKTVLVLCLLIAAVYS
RPDTYDTRYDNFDVESLVENVRLLKSYAHCFLSTGPCTPEGTAFKKTIPDALQSGCSKCSP
RQRHLVRVVVKGFQSKTPDLWQQLVKKEDPNGEYKATFTKFINASD
>AlCSP12
MKACIALCVLSVAVMALA
RPEDSQYTDRYDNVNLDEILSNRRLLTPYVKCLLDQGKCAPDAKELKEHIWEALENECGK
CSEKQRKGTRRVISHLINNEEDYWNELTAKYDPERKFTAKYEKELKEINA
>AlCSP13
MRNWLLCLCVLTVVVSCYS
QGPNRYENFNTDAIIQNDRILLAYYKCVMDKGPCTRDGKNFKRVLPETLATACGRCNPKQ
KTIVRTLLLGIRSKSEPRFLELLDKYSPDRSNRDALYTFLVTGN
>AlCSP14
RTLAPLVLRGACPQCSMQETRQIRRTLAFVQRNYPWEWAKIVRQYG
>AlCSP15
MRVLIVLSCMLVVAFA
AEKYNAKYDNFDVETLISNDRLLKAYINCFLDKGRCTPEGSDFKKTLPEAIETTCAKCTEK
QKGNIRKVIKAIQQKHPKEWDALVKKNDPSGKNRVNFDKFIQGSS
>AlCSP16
MIENKRSFRVSLIFTYIFLVTVLAQEKYYDRRYDYYEIDSLIQNRRLLKKYLDCFLGKGPCT
PIGKVFKQILPEAVATACKKCSPSQRRLARKAFNAFDRFFPDTYVEFVHKLDPKNKYYEAF
ENAITNA
>AlCSP17
MNSFIVLCLFGLVAVSLA
RPDSTYTNRYDNVNLNEILSNRRLLVPYIKCILDQGKCTPEGKELKSHIKEALEEDCAKCT
PTQRDGTRQVMGHLINHETDYWNQLKAKYDPQSKYASKHEQELRTLKN
>AlCSP18
MKCIYVLSLLLAFVAVQA
EDKYSTENDDLDIDAVVADLDTLKGFLGCFMDTVTCPAVPADFKKDIPEAVKTNCSKCTD
AQKHIFHKFLLGLKEKLPSDYEAFKKKFDPENKHFTALEAAVASF
>AlCSP19
MKIVLVTLCLALGVLA
EEQYGSANDDFDISEVLHNERLLQAYGRCLLDKGPCTAEVKTLKEKLPEALETRCAKCTE
KQKQMGKALAQEVKKNHPDLWKELVAHYDPEGKYQEAWKEFLKE
>AlCSP20
MQIKYALVLCCVAATAVA
QTQRPAVSDTALEDALQDKRFIQRQLKCALGEAPCDPIGKRLKTLAPLVLRGACPQCTPQE
TKQIQRTLSYVQRNFPQQWAKIVRQYAG
