# Supplementary material for: Molecular identification and expression patterns of odorant binding protein and chemosensory protein genes in Athetis lepigone (Lepidoptera: Noctuidae)
Source: PeerJ. 2017 Mar 30;5:e3157. doi: 10.7717/peerj.3157 (PMC5376112; doi:10.7717/peerj.3157)
Supplement: Figure S1 [file peerj-05-3157-s004.pdf]

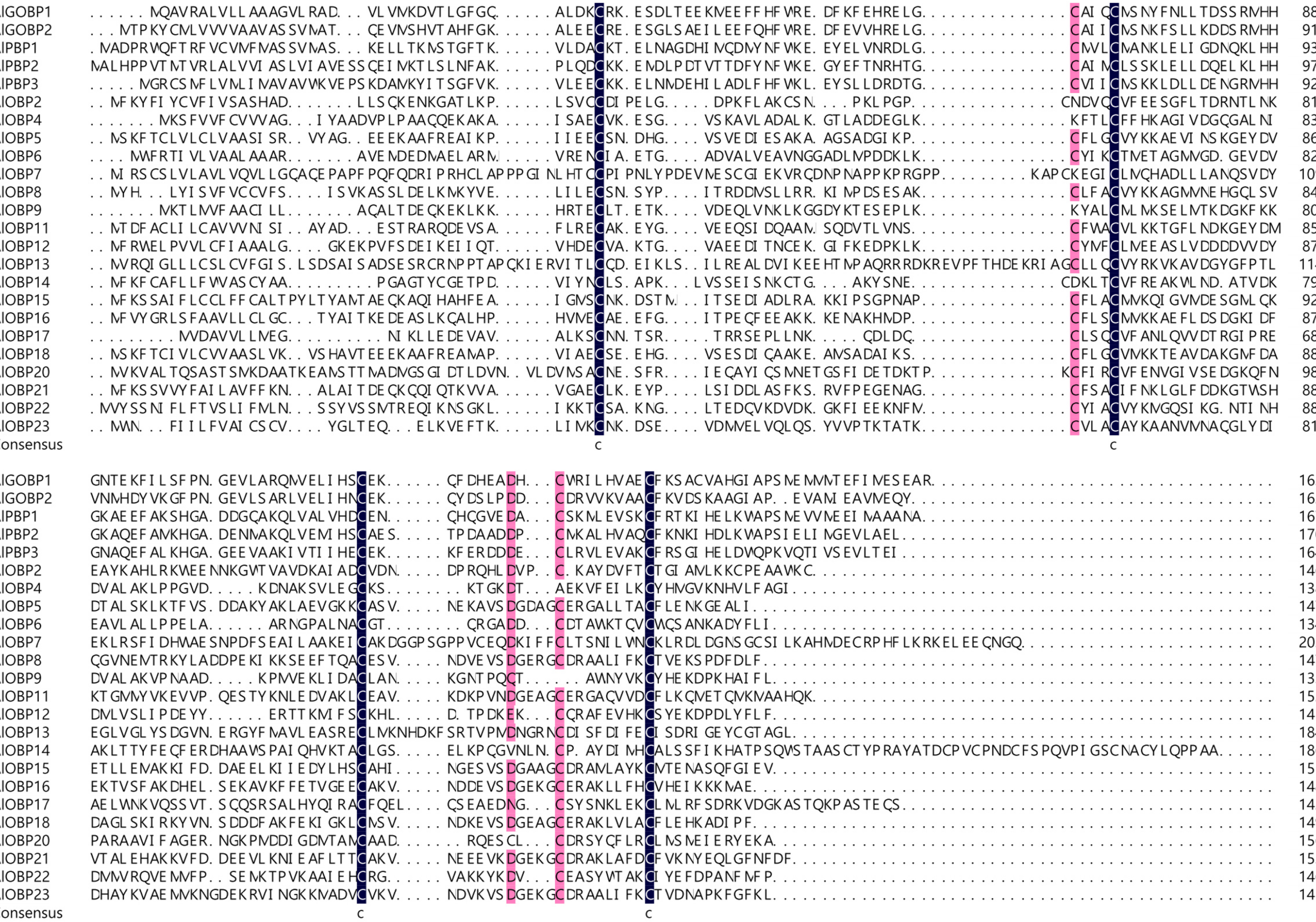
IGOBP1 ......MQAVRALVLLAAAGVLRAD...VLVMKDVTLGFGC.....ALDKCRK.ESDLTEEKMEEFFHFWRE.DFKFEHRELG..............CAICCMSNYFNLLTDSSRMHH 88
IGOBP2 ...MTPKYCMLVVVVAAVASSVMAT..CEVMSHVTAHFGK.....ALEECRE.ESGLSAEILEEFQHFWRE.DFEVVHRELG..............CAIICMSNKFSLLKDDSRMHH 91
IPBP1 .MADPRWQFTRFVCVMFMASSVMAS...KELLTKMSTGFTK.....VLDACKT.ELNAGDHIMCDMYNFWKE.EYELVNRDLG..............CMVLCMANKLELIGDNQKLHH 93
IPBP2 MALHPPVTMTVRLALVVIASLVIAVESSCEIMKTLSLNFAK.....PLQDCKK.EMDLPDTVTTDFYNFWKE.GYEFTNRHTG..............CAIMCLSSKLELLDQELKLHH 97
IPBP3 ....MGRCSMFLVMLIMAVAVWKVEPSKDAMKYITSGFVK.....VLEECKK.ELNMDEHILADLFHFWKL.EYSLLDRDTG..............CVIICMSKKLDLLDENGRMHH 92
IOBP2 ..MFKYFIYCVFIVSASHAD.......LLSCKENKGATLKP.....LSVCCDIPELG....DPKFLAKCSN....PKLPGP...........CNDVCCVFEESGFLTDRNTLNK 81
IOBP4 ......MKSFVVFCVVVAG...IYAADVPLPAACQEKAKA.....ISAECVK.ESG....VSKAVLADALK.GTLADDEGLK..............KFTLCFFHKAGIVDGCGALNI 83
IOBP5 ..MSKFTCLVLCLVAASISR..VYAG..EEEKAAFREAIKP.....IIEECSN.DHG....VSVEDIESAKA.AGSADGIKP..............CFLGCVYKKAEVINSKGEYDV 86
IOBP6 ....MMFRTIVLVAALAAAR.......AVEMDEDMAELARM.....VRENCIA.ETG....ADVALVEAVNGGADLMPDDKLK..............CYIKCTMETAGMVGD.GEVDV 82
IOBP7 ..MIRSCSLVLAVLVQVLLGCACEPAPFPQFQDRIPRHCLAPPGINLHTCCPIPNLYPDEVMESCGIEKVRCDNPNAPPKPRGPP..........KAPCKEGICLMCHADLLLANQSVDY 10
IOBP8 ..MYH...LYISVFVCCVFS....ISVKASSLDELKMKYVE.....LILECSN.SYP....ITRDDMSLLRR.KIMPDSESAK..............CLFACVYKKAGMVNEHGCLSV 84
IOBP9 ......MKTLMVFAACILL.......ACALTDECKEKLKK.....HRTECLT.ETK....VDEQLVNKLKGGDYKTESEPLK..............KYALCMLMKSELMTKDGKFKK 80
IOBP11 ..MTDFACLILCAVVVNISI..AYAD...ESTRARQDEVSA.....FLRECAK.EYG....VEEQSIDQAAM.SQDVTLVNS..............CFWACVLKKTGFLNDKGEYDM 85
IOBP12 ..MFRWELPVVLCFIAAALG....GKEKPVFSDEIKEIIQT.....VHDECVA.KTG....VAEEDITNCEK.GIFKEDPKLK..............CYMFCLMEEASLVDDDDVVDY 87
IOBP13 ..MVRQIGLLLCSLCVFGIS.LSDSAISADSESRCRNPPTAPCKIERVITLCCD.EIKLS..ILREALDVIKEEHTMPAQRRRDKREVPFTHDEKRIAGCLLCCVYRKVKAVDGYGFPTL 11
IOBP14 ..MFKFCAFLLFWVASCYAA.........PGAGTYCGETPD.....VIYNCLS.APK...LVSSEISNKCTG....AKYSNE...........CDKLTCVFREAKWLND.ATVDK 79
IOBP15 ..MFKSSAIFLCCLFFCALTPYLTYAMTAECKAQIHAHFEA.....IGMSCNK.DSTM..ITSEDIADLRA.KKIPSGPNAP..............CFLACMMKQIGVMDESGMLCK 92
IOBP16 ..MFVYGRLSFAAVLLCLGC...TYAITKEDEASLKCALHP.....HVMECAE.EFG....ITPECFEEAKK.KENAKHMDP..............CFLSCMMKKAEFLDSDGKIDF 87
IOBP17 .........MVDAVVLLMEG..........NIKLLEDEVAV.....ALKSCNN.TSR.....TRRSEPLLNK.....CDLDC..............CLSCCVFANLQVVDTRGIPRE 68
IOBP18 ..MSKFTCIVLCVVAASLVK..VSHAVTEEEKAAFREAMAP.....VIAECSE.EHG....VSESDICAAKE.AMSADAIKS..............CFLGCVMKKTEAVDAKGMFDA 88
IOBP20 ..MVKVALTQSASTSMKDAATKEAMSTTMADMGSGIDTLDVN..VLDVMSACNE.SFR....IECAYICSMNETGSFIDETDKTP............KCFIRCVFENVGIVSEDGKQFN 98
IOBP21 ..MFKSSVVYFAILAVFFKN...ALAITDECKCQIQTKVVA.....VGAECLK.EYP....LSIDDLASFKS.RVFPEGENAG..............CFSACIFNKLGLFDDKGTWSH 88
IOBP22 .MVYSSNIFLFTVSLIFMLN...SSYVSSMTREQIKNSGKL.....IKKTCSA.KNG....LTEDCVKDVDK.GKFIEEKNFM..............CYIACVYKMGQSIKG.NTINH 88
IOBP23 ..MMN...FIILFVAICSCV....YGLTEQ...ELKVEFTK.....LIMKCNK.DSE....VDMVELVQLQS.YVVPTKTATK..............CVLACAYKAANVMNACGLYDI 81
onsensus c c
IGOBP1 GNTEKFILSFPN.GEVLARQMVELIHSCEK......CFDHEADH...CWRILHVAECFKSACVAHGIAPSMEMVMTEFIMESEAR............ 16
IGOBP2 VNMHDYVKGFPN.GEVLSARLVELIHNCEK......CYDSLPDD...CDRVVKVAACFKVDSKAAGIAP..EVAMIEAVMEQY............. 16
IPBP1 GKAEEFAKSHGA.DDGCAKQLVALVHDCEN......CHCGVEDA...CSKMLEVSKCFRTKIHELKWAPSMEVVMEEIMAAANA............ 16
IPBP2 GKAQEFAMKHGA.DENMAKQLVEMIHSCAES.....TPDAADDP...CMKALHVAQCFKNKIHDLKWAPSIELIMGEVLAEL............. 17
IPBP3 GNAQEFALKHGA.GEEVAAKIVTIIHECEK......KFERDDDE...CLRVLEVAKCFRSGIHELDWQPKVQTIVSEVLTEI............. 16
IOBP2 EAYKAHLRKWEENNKGWTVAVDKAIADCVDN.....DPRQHLDVP..C.KAYDVFTCTGIAMLKKCPEAAWKC............... 14
IOBP4 DVALAKLPPGVD.....KDNAKSVLEGCKS.......KTGKDT...AEKVFEILKCYHMGVKNHVLFAGI.............. 13
IOBP5 DTALSKLKTFVS.DDAKYAKLAEVGKKCASV.....NEKAVSDGDAGCERGALLTACFLENKGEALI............ 14
IOBP6 EAVLALLPPELA......ARNGPALNACGT.......CRGADD...CDTAWKTCVCWCSANKADYFLI.......... 13
IOBP7 EKLRSFIDHWAESNPDFSEAILAAKEICAKDGGPSGPPVCEQDKIFFCLTSNILWNCKLRDLDGNSGCSILKAHMDECRPHFLKRKELEECNGQ...... 20
IOBP8 CGVNEMTRKYLADDPEKIKKSEEFTQACESV.....NDVEVSDGERGCDRAALIFKCTVEKSPDFDLF........ 14
IOBP9 DVALAKVPNAAD.....KPMVEKLIDACLAN.....KGNTPQCT.....AWNYVKCYHEKDPKHAIFL....... 13
IOBP11 KTGMMYVKEVVP.QESTYKNLEDVAKLCEAV.....KDKPVNDGEAGCERGACVVDCFLKCMETCMKMAAHQK..... 15
IOBP12 DMLVSLIPDEYY......ERTTKMIFSCKHL.....D.TPDKEK...CCRAFEVHKCSYEKDPDLYFLF..... 14
IOBP13 EGLVGLYSDGVN.ERGYFMAVLEASRECLMKNHDKFSRTVPMDNGRNCDISFDIFECISDRIGEYCGTAGL..... 18
IOBP14 AKLTTYFECFERDHAAWSPAIQHVKTACLGS.....ELKPCGVNLN.CP.AYDIMHCALSSFIKHATPSQWSTAASCTYPRAYATDCPVCPNDCFSPQVPIGSCNACYLQPPAA...... 18
IOBP15 ETLLEMAKKIFD.DAEELKIIEDYLHSCAHI.....NGESVSDGAAGCDRAMLAYKCMTENASQFGIEV...... 15
IOBP16 EKTVSFAKDHEL.SEKAVKFFETVGEECAKV.....NDDEVSDGEKGCERAKLLFHCVHEIKKMAE...... 14
IOBP17 AELWNKVQSSVT.SCQSRSALHYQIRACFQEL....CSEAEDNG...CSYSNKLEKCLMLRFSDRKVDGKASTQKPASTECS..... 14
IOBP18 DAGLSKIRKYVN.SDDDFAKFEKIGKLCMSV.....NDKEVSDGEAGCERAKLVLACFLEHKADIPF...... 14
IOBP20 PARAAVIFAGER.NGKPMDDIGDMTAMCAAD......RQESCL...CDRSYCFLRCLMSMEIERYEKA...... 15
IOBP21 VTALEHAKKVFD.DEEVLKNIEAFLTTCAKV.....NEEEVKDGEKGCDRAKLAFDCFVKNYEQLGFNFDF...... 15
IOBP22 DMMVRQVEMVFP..SEMKTPVKAAIEHCRG......VAKKYKDV...CEASYWTAKCIYEFDPANFMFP...... 14
IOBP23 DHAYKVAEMVKNGDEKRVINGKKMADVCVKV.....NDVKVSDGEKGCDRAALIFKCTVDNAPKFGFKL...... 14
onsensus c c
