# Supplementary material for: Molecular identification and expression patterns of odorant binding protein and chemosensory protein genes in Athetis lepigone (Lepidoptera: Noctuidae)
Source: PeerJ. 2017 Mar 30;5:e3157. doi: 10.7717/peerj.3157 (PMC5376112; doi:10.7717/peerj.3157)
Supplement: Figure S2 [file peerj-05-3157-s005.pdf]

```
AlepCSP3    ........MNALLVAVFALVASSA.........LAYDEIYDKIDVDKILGDDALFSAYINCMLDKGDCSVEHSADFRKLLPEVIATSCAKCSPIQRQNVRKTVKALSEKRPDEFAEFRTKY   104
AlepCSP4    ........MRAVLLMCVFVYAVVAQDVNDMNLPKYDSRYDYLDVDAIFTNKRLVRNYVDCLINAVRCTPEG.KALKRILPEALRTKCVRCTERQCRTAVKVIKRLKNEYPDEWAKLSSRW   112
AlepCSP6    ........MHSTIAMLLLVYLTICSNATE...TSTYTTKYDGIDLDEILNNDRLLTGYVNCLLDIGPCTADG.KELKCNLPDAIENDCKKCTEKQREGSERVMHYIIDNRPDDWVKLEDKY   109
AlepCSP7    ........MKVLVVLPVLVAFAAAAAAELS...PAELSMLEAFDYETLLANKELSCKLFDCMLEKGDCGEYK..QVADLSMKTLQSKCAECTPAQKAKYENVLKQLKEKYEPVYNELLKKA   108
AlepCSP8    ........MKVIVAIALLCIVAVAWGKPA...G.TYTDKWDHINVDEVLESCRLLKAYVDCLMDRGRCTPDG.KALKETLPDALEHECSKCTEKQKTGSDKVIRHLVNKRPDLWKELSTKY   108
AlepCSP9    ........MKFVLLLCVMVAAVLAED.........KYTDKYDNLNVDEILTNKRLLEAYVNCVLEKGKCTAEG.KELKEHLQDAIETGCKKCTEAQEKGACKVIDYLIKNELGYWRELTDKY   104
AlepCSP10   ........MNYLVLSVVVTLAAFVAAET.......YTDRYDHINIDEIIDNRKLLVPYIKCTLDQGRCTPEG.RELKAHIKDAMQTSCSKCTPKQRKAARKVVKHIRAKEQDYWKCIIAKY   105
AlepCSP11   ........MKTVLVLCLLIAAVYSR..P.....DTYDTRYDNFDVESLVENVRLLKSYAHCFLSTGPCTPEG.TAFKKTIPDALQSGCSKCSPRQRHLVRVVVKGFQSKTPDLWQCLVKKE   105
AlepCSP12   ........MKACIALCVLSVAVMALARPE...DSQYTDRYDNVNLDEILSNRRLLTPYVKCLLDQGKCAPDA.KELKEHIWEALENECGKCSEKQRKGTRRVISHLINNEEDYWNELTAKY   109
AlepCSP13   .......MRNWLLCLCVLTVVVSCYSQG........PNRYENFNTDAIIQNDRILLAYYKCVMDKGPCTRDG.KNFKRVLPETLATACGRCNPKQKTIVRTLLLGIRSKSEPRFLELLDKY   105
AlepCSP15   ........MRVLIVLSCMVVAFA...A.....EKYNAKYDNFDVETLISNDRLLKAYINCFLDKGRCTPEG.SDFKKTLPEAIETTCAKCTEKQKGNIRKVIKAIQCKHPKEWDALVKKN   104
AlepCSP16   MIENKRSFRVSLIFTYIFLVTVLAC.E.....KYYDRRYDYYEIDSLIQNRRLLKKYLDCFLGKGPCTPIG.KVFKCILPEAVATACKKCSPSQRRLARKAFNAFDRFFPDTYVEFVHKL   113
AlepCSP17   ........MNSFIVLCLFGLVAVSLARP...DSTYTNRYDNVNLNEILSNRRLLVPYIKCILDQGKCTPEG.KELKSHIKEALEEDCAKCTPTQRDGTRQVMGHLINHETDYWNCLKAKY   108
AlepCSP18   ........MKCIYVLSLLLAFVAVQAED......KYSTENDDLDIDAVVADLDTLKGFLGCFMDTVTCPAVP.ADFKKDIPEAVKTNCSKCTDAQKHIFHKFLLGLKEKLPSDYEAFKKKF   106
AlepCSP19   ........MKIVLVTLCLALGVLAEEQ........YGSANDDFDISEVLHNERLLCAYGRCLLDKGPCTAEV.KTLKEKLPEALETRCAKCTEKQKQMGKALACEVKKNHPDLWKELVAHY   104
AlepCSP20   .....MQIKYALVLCCVAATAVAQTQR..........PAVSDTALEDALCDKRFICRCLKCALGEAPCDPIG.KRLKTLAPLVLRGACPQCTPCETKCICRTLSYVQRNFPCQWAKIVRCY   105
Consensus                                                          c         c                   c  c

AlepCSP1    DRDGKFARKYED..ELRTVA..........................................................................   124
AlepCSP2    DPEGKHKEGLKSFLEKYA............................................................................   124
AlepCSP3    DPKGEYEKSFTEFVMGTD............................................................................   122
AlepCSP4    DPTGDFTRYFEEFLAKENFNTIPGSGSAIPTSSPLVPPRVTTMPATVATPAPGPTEPAPACPAIFNRFGDDDEVMMGSPSSAGMTPRPMTCATTRPTTTMRPTTTMRPITSKPLSPRPTM   232
AlepCSP6    NTDGSYKLKYLASKLTEADKETNVTTSEENTKNVSKESSKE.....................................................   150
AlepCSP7    GATQKT........................................................................................   114
AlepCSP8    DPDNIYCERYKN..KIEAVKC.........................................................................   127
AlepCSP9    DPTGTWRKTYEDR.AKA.......AGIKIPE...............................................................   127
AlepCSP10   DPNDEYKENYET..FLETTD..........................................................................   123
AlepCSP11   DPNGEYKATFTKFINASD............................................................................   123
AlepCSP12   DPERKFTAKYEK..ELKEINA.........................................................................   128
AlepCSP13   SPDRSNRDALYTFLVTGN............................................................................   123
AlepCSP15   DPSGKNRVNFDKFICGSS............................................................................   122
AlepCSP16   DPKNKYYEAFENAITNA.............................................................................   130
AlepCSP17   DPCSKYASKHEQ..ELRTLKN.........................................................................   127
AlepCSP18   DPENKHFTALEAAVASF.............................................................................   123
AlepCSP19   DPEGKYCEAWKE..FLKE............................................................................   120
AlepCSP20   AG............................................................................................   107
Consensus

AlepCSP1    ..............................................................   124
AlepCSP2    ..............................................................   124
AlepCSP3    ..............................................................   122
AlepCSP4    MTWSGAATNTCPTRFPLRPVAELPIPYSTAITLIDQIGYKIIKTTELVTDLLKHTVRAVVGR   294
AlepCSP6    ..............................................................   150
AlepCSP7    ..............................................................   114
AlepCSP8    ..............................................................   127
AlepCSP9    ..............................................................   127
AlepCSP10   ..............................................................   123
AlepCSP11   ..............................................................   123
AlepCSP12   ..............................................................   128
AlepCSP13   ..............................................................   123
AlepCSP15   ..............................................................   122
AlepCSP16   ..............................................................   130
AlepCSP17   ..............................................................   127
AlepCSP18   ..............................................................   123
```
